# Potentiation of Brain-Derived Neurotrophic Factor-Induced Protection of Spiral Ganglion Neurons by C3 Exoenzyme/Rho Inhibitor

**DOI:** 10.3389/fncel.2021.602897

**Published:** 2021-03-11

**Authors:** Jennifer Harre, Laura Heinkele, Melanie Steffens, Athanasia Warnecke, Thomas Lenarz, Ingo Just, Astrid Rohrbeck

**Affiliations:** ^1^Department of Otorhinolaryngology, Head and Neck Surgery, Hannover Medical School, Hannover, Germany; ^2^Cluster of Excellence “Hearing4all” of the German Research Foundation (EXC 2177/1), Hannover, Germany; ^3^Institute of Toxicology, Hannover Medical School, Hannover, Germany

**Keywords:** spiral ganglion neurons, RhoA, C3 exoenzyme, brain-derived neurotrophic factor, neuroprotection

## Abstract

Preservation of the excitability of spiral ganglion neurons (SGN) may contribute to an improved speech perception after cochlear implantation. Thus, the application of exogenous neurotrophic factors such as the neurotrophin brain-derived neurotrophic factor (BDNF) to increase SGN survival *in vitro* and *in vivo* is a promising pharmacological approach in cochlear implant (CI) research. Due to the difficult pharmacokinetic profile of proteins such as BDNF, there is a quest for small molecules to mediate the survival of SGN or to increase the efficacy of BDNF. The C3 exoenzyme from *Clostridium botulinum* could be a potential new candidate for the protection and regeneration of SGN. Inhibition of the RhoA GTPase pathway which can be mediated by C3 is described as a promising strategy to enhance axonal regeneration and to exert pro-survival signals in neurons. Nanomolar concentrations of C3, its enzymatically inactive form C3^E174Q^, and a 26mer C-terminal peptide fragment covering amino acid 156–181 (C3^156-181^) potentiated the neuroprotective effect on SGN mediated by BDNF *in vitro*. The neuroprotective effect of C3/BDNF was reduced to the neuroprotective effect of BDNF alone after the treatment with wortmannin, an inhibitor of the phosphatidylinositol-3-kinase (PI3K).The exoenzyme C3 (wild-type and enzyme-deficient) and the C3 peptide fragment C3^154–181^ present novel biologically active compounds for the protection of the SGN. The exact underlying intracellular mechanisms that mediate the neuroprotective effect are not clarified yet, but the combination of BDNF (TrkB stimulation) and C3 exoenzyme (RhoA inhibition) can be used to protect SGN *in vitro*.

## Introduction

According to the World Health Organization, over 460 million people worldwide suffer from hearing loss (WHO, 2020). The most common form is the sensorineural hearing loss caused by irreversible damage to the inner hair cells, followed by the degeneration of the spiral ganglion neurons (SGN). The hair cell loss is compensated with the aid of a cochlear implant (CI) which is an electrode array that directly stimulates the residual SGN enabling the transport of acoustic signals to the brain (Lenarz, 2017; Perenyi et al., 2018).

However, the effectiveness of CIs varies among patients and is limited by the excitability and the number of residual SGN. Missing neurotrophic and electrical support can be the primary cause of SGN degeneration. Thus it is therapeutically desirable to preserve healthy and to regenerate damaged neurons by neuroprotective factor application. Several neuroprotective factors have been identified that regulate neuronal differentiation, survival, and also axonal outgrowth (Wefstaedt et al., 2005; Schmidt et al., 2018). The most intensively studied factor in the field of hearing research is the brain-derived neurotrophic factor (BDNF). Several *in vitro* and *in vivo* studies showed that exogenous application of BDNF increased the survival of SGN and further also enhanced the protective effects of electrical stimulation of the auditory nerve (McGuinness and Shepherd, 2005; Warnecke et al., 2007; Agterberg et al., 2009). In deafened guinea pigs, even a belated treatment with BDNF increased the survival of SGN (Agterberg et al., 2009; Shibata et al., 2011). The neuroprotective effect of BDNF is mediated by the binding to the high-affinity tyrosine kinase receptor B (TrkB), which is expressed in the SGN. The neuroprotective effect of BDNF can be further enhanced by the administration of a cocktail of different factors (Kaiser et al., 2013; Kranz et al., 2014; Schwieger et al., 2015; Stolle et al., 2017). Up to now, there is no approved drug for clinical application (Schilder et al., 2019). Therefore, it is necessary to identify alternative substances instead of or in combination with BDNF which are pharmacologically easier to handle and are more suitable for clinical use. A novel candidate for the protection of SGN is the C3 exoenzyme (C3) derived from *Clostridium botulinum*. C3 is a 24 kDa single-chain protein, belongs to the family of ADP-ribosyltransferases, and functions as a selective inhibitor of the small Rho guanosine triphosphate hydrolases RhoA, B, and C (Chardin et al., 1989; Sekine et al., 1989; Schmidt, 2018). The Rho GTPase hydrolyzes GTP to GDP and thus in the active state GTP is bound to RhoA whereas in the inactive state GDP is bound (Etienne-Manneville and Hall, 2002; Hodge and Ridley, 2016; Kalpachidou et al., 2019). The C3 inactivates the Rho GTPase *via* ADP-ribosylation at the amino acid asparagine at position 41 (Sekine et al., 1989; Schmidt, 2018). Inactivation of Rho leads to functional changes in the actin cytoskeleton, which is associated with morphological changes such as the rounding up of most mammalian cells (Paterson et al., 1990; Rohrbeck et al., 2012). The Rho proteins regulate many other essential cellular functions such as proliferation, apoptosis, formation and maintenance of dendritic spines, and especially the neuronal growth cone (Leemhuis et al., 2007; Huelsenbeck et al., 2012; Schröder et al., 2015). For example, primary murine hippocampal neurons react with an increased axonal outgrowth after treatment with nanomolar concentrations of C3 (Ahnert-Hilger et al., 2004). After spinal cord injury, C3 promotes axonal repair mechanisms (Ruff et al., 2008). Moreover, C3 promotes the outgrowth of dorsal root ganglions *in vitro* (Fournier et al., 2003; Auer et al., 2012; Quarta et al., 2017). In contrast, activation of RhoA and its downstream protein kinase ROCK induced neuronal apoptosis (Zhang et al., 2010). However, BDNF protects cortical neurons from phenylalanine-induced apoptosis by inactivation of Rho (Zhang et al., 2010). Since the C3 transferase can inactivate Rho GTPases, it is used for neuronal regeneration (Auer et al., 2012; Tan et al., 2018a). Despite being acknowledged as a potent neuroprotective agent, the effect and function of C3 have not been analyzed in the auditory system, especially not in SGN up to now.

In this study, the effect of different constructs of C3 on SGN alone and in combination with BDNF is investigated. The SGN are the auditory neurons of the inner ear connecting the hair cells with the auditory cortex and are a prerequisite for auditory perception. We performed an *in vitro* study with primary neonatal rat SGN, a common serum-deprivation model for the investigation of potential effects of novel neuroprotective factors on auditory neurons (Kranz et al., 2014; Schwieger et al., 2015; Schulze et al., 2020). After dissociation and seeding of the SGN, the neurons display no protruding processes and consist of the soma only. Sporadic neurite regrowth is observed within 48 h in the SGN medium. In the presence of adequate stimuli (e.g., BDNF), however, a significant increase in the outgrowth of neurites from the auditory neurons can be observed *in vitro*. Since the underlying intracellular signaling mechanism of C3 is not clarified yet, we used different inhibitors for its identification.

## Materials and Methods

### Spiral Ganglion Neuron Isolation and Cultivation

For the spiral ganglion neuron (SGN) dissection, *n* = 16 neonatal Sprague-Dawley rats of both sexes (postnatal day 3–5) were used for each preparation following the European Communities Council Directive 2010/63/EU for the protection of animals used for experimental purposes and the German law for animal protection. The local authorities (Zentrales Tierlaboratorium, Laboratory Animal Science, Hannover Medical School) approved this procedure (no.: 2016/118) and as demanded by law, the experiments were reported regularly. For the exclusive sacrifice of animals for tissue analysis in research, no further approval is needed if no other treatment is applied beforehand (§4). After decapitation, the skull was opened along the midline, the brain tissue was removed and the two head halves of the skull base were isolated and immersed in ice-cold Phosphate-Buffered Saline (PBS; 0.172 mg/ml, PBS tablets, Gibco by Life Technologies). Further dissection was performed under microscopic view (Leica MZ-6, Bensheim, Germany) by using Dumont Mirror Finish Forceps (#5, Fine Science Tool, Heidelberg, Germany). The bony cochlea capsule was carefully opened and the membranous cochlea was obtained. In the following, the stria vascularis and the organ of Corti were removed. Finally, the entire spiral ganglion was removed from the modiolus and was placed in ice-cold Ca^2+^/Mg^2+^-free Hank’s balanced salt solution (HBSS, Invitrogen). According to Lefebvre et al. (1991) and Hegarty et al. (1997) the enzymatic and mechanical dissociation of the spiral ganglia were performed: therefore, the spiral ganglia (*n* = 16) were incubated for 16 min at 37°C in 2 ml Ca^2+^/Mg^2+^-free HBSS containing 0.1% Trypsin (Serva) and 0.01% DNase l (Roche). Enzymatic dissociation was stopped by adding 200 μl FBS (Invitrogen). Following this, the spiral ganglion cell clusters were rinsed three times in a culture medium. Then, the mechanical dissociation was performed by using three different pipettes in descending sizes, each 90–100 times. Finally, 10 μl of trypan blue (Sigma–Aldrich, Taufkirchen, Germany) was given to 10 μl of the cell suspension for counting the viable cells (glial cells, fibroblasts, and neurons) in a Neubauer cytometer (Brandt).

### Spiral Ganglion Medium

The serum-free culture medium was composed of Panserin 401 (PAN, Biotech, Aidenbach, Germany) and was supplemented with 4-(2-hydroxyethyl)-1-piperazineethanesulfonic acid (HEPES; 23.43 mM; Invitrogen, Karlsruhe, Germany), PBS (0.172 mg/ml, PBS tablets, Gibco by Life Technologies), penicillin (30 U/ml; Biochrom, Germany) and glucose (0.15%; Braun, Melsungen, Germany). At last, N2-supplement (0.1 μg/ml; Invitrogen) and insulin (8.7 μg/ml; Biochrom, Germany) were added. For some wells, BDNF (50 ng/ml) was added to the culture medium.

### Coating of the Plates

For the coating of a 96-well microtiter plate (TPP), 50 μl of poly-D/L-ornithine (0.1 mg/ml in PBS; Sigma–Aldrich) was given at ambient temperature into each well. After 1 h, the solution was removed and each well was rinsed with PBS. Then the plate was incubated with 50 μl of the laminin solution (0.01 mg/ml in PBS; natural from mouse, Life Technologies, Carlsbad, CA, USA) for 1 h at 37°C, 5% CO_2_ in a humidified atmosphere. Finally, the solution was removed and each well was rinsed with and stored in PBS until the cell seeding was performed (Wefstaedt et al., 2005).

### C3 Proteins

We have used the full-length C3 exoenzyme, its enzymatically inactive form C3^E174Q^, and a 26mer C-terminal peptide fragment covering amino acid 156–181 (C3^156-181^) of C3. The expression and purification of the recombinant C3 protein and the C3^156-181^ synthesis were carried out as described previously (Rohrbeck et al., 2015a). Briefly, C3 and C3^E174Q^ were expressed as recombinant GST-fusion protein in *E. coli* TG1 using the pGEX-2T vector system and purified by affinity chromatography using glutathione-sepharose. C3 was mobilized from the glutathione-sepharose by thrombin digest. Thrombin was removed by precipitation with benzamidine-sepharose beads (AP Biosciences, New York, NY, USA). The activity of the C3 exoenzyme was measured by an *in vitro* ADP-ribosylation assay. C3^154–182^ was synthesized at IPF PharmaCeuticals GmbH (Hannover, Germany). The lyophilized peptide was reconstituted in PBS pH 7.5, sterile filtered (0.22 μm), and used for the experiments as indicated.

### Initial Screening Experiments of C3 Constructs and SGN

In initial screening experiments, we determined the concentrations of C3 constructs and the incubation time for further experimental settings. For those cell culture experiments, the SGN were isolated and cultivated as described before. Following, the SGN incubation was performed with various concentrations at 1 nM, 20 nM, 50 nM, and 100 nM of C3, C3^E174Q^, and C3^156-181^. The negative control did not receive any additional growth factors whereas a positive control for SGN survival, was supplemented with BDNF (50 ng/ml, Invitrogen, Karlsruhe, Germany). Incubation took place over 48 h. After incubation, the SGN cultures got fixed with Methanol/Acetone 1:1 and immunocytochemical determination took place as described in the following.

### Co-cultivation of SGN With Different C3 Constructs and Inhibitors

After cell counting, the viable cells were seeded at a density of 1*10^4^ cells/well, in a total volume of 100 μl/well. For the first part, the experimental setting is briefly described as followed: 33 wells of a 96-well microtiter plate were seeded with cells, each with 50 μl of the cell suspension and 50 μl of the following factors: (i) *n* = 3 wells were supplemented with C3 wild-type (C3); (ii) *n* = 3 wells contained the enzymatically inactive mutant C3^E174Q^; and (iii) *n* = 3 wells contained the C3- derived 26mer peptide C3^156-181^ each one of them at a concentration of 50 nM. For determining the concentrations to be applied, initial screening experiments were performed and previous studies were used for an orientation (Höltje et al., 2009; Loske et al., 2012). For another *n* = 12 wells, the setting was repeated, except that the additional growth factor BDNF was supplemented to the culture medium. Therefore, we included: (iv) *n* = 3 wells supplemented with C3 and BDNF; (v) *n* = 3 wells with C3^E174Q^ and BDNF; (vi) *n* = 3 wells with C3^156-181^ and BDNF. As a positive control for SGN survival, (vii) *n* = 3 wells were supplemented with 50 μl of BDNF (50 ng/ml, recombinant human BDNF, Invitrogen, Karlsruhe, Germany), whereas the negative control of (viii) *n* = 3 wells did not receive any additional growth factors. To determine the survival rate; and (ix) *n* = 3 wells did only receive culture medium and were fixed after 4 h.

Subsequently, further tests were performed in combination with five selected inhibitors to verify the intracellular pathway through which the neuroprotective effect of C3 is mediated. Therefore, 27 wells of a 96–well microtiter plate were seeded with cells and treated with C3 (50 nM) in combination with BDNF (50 ng/ml) and additionally with an inhibitor to reverse the effect of C3. For the inhibition, we used:

‐ AG490 (#658401; Calbiochem, Merck Millipore) reversible inhibiting Jak2 and several other kinase signaling pathways.
‐ Wortmannin (#12-338; Calbiochem, Merck Millipore) specific inhibitor of the phosphatidylinositol-3-kinase (PI3K).
‐ SB203580 (#559389; Calbiochem, Merck Millipore) inhibits the p38MAPK.
‐ Y-27632 (Calbiochem, Merck Millipore) inhibitor of Rho-associated, coiled-coil containing protein kinase (ROCK).
‐ NSC23766 (Calbiochem, Merck Millipore) inhibitor of Rho-GTPase Rac1.

The inhibitors AG490, wortmannin, and SB203580 were diluted in dimethyl sulfoxide (DMSO, AppliChem, Darmstadt, Germany) and Y-27632 and NSC23766 were diluted in aqua dest. The final concentrations were 100 nM for AG490 and SB203580, 5 nM for wortmannin, 10 μM for Y-27632, and 75 μM for NSC23766.

Spiral ganglion cell cultivation was maintained at 37°C, 5% CO_2_, and in a humidified atmosphere. After 4 h of cultivation, the first three wells were fixed with methanol/acetone (1:1) and the number of seeded SGN was determined as followed and is noted as “seeding control.” The remaining wells were cultured for 48 h in total and fixation was performed with a 1:1 solution of methanol and acetone for 10 min at room temperature. Afterward, cells were washed three times with PBS. Altogether, the experimental settings were repeated three times resulting in a total of *n* = 9 per experimental condition.

### Immunocytochemical Staining

For the identification of SGN in the dissociated spiral ganglion cell culture (as spiral ganglia contain different cell types such as glial cells, neurons, and fibroblasts), neuron-specific staining was necessary, which was performed *via* the heavy chain 200 kDa neurofilament. To perform the immunocytochemistry, different solutions of the VECTASTAIN^®^ Elite^®^ ABC Kit (Vector Laboratories, Burlingame) were sequentially used for each well, briefly described as followed. First, a dilution (1:500) of the monoclonal anti-mouse neurofilament-antibody (200 kDa, clone RT97, Novocastra Limited, Newcastle upon Tyne, UK) was performed in PBS containing 1.5% normal horse serum (Vectastain Elite ABC-Kit, Vector Labs, Burlingame, CA, USA) and was incubated for 1 h at 37°C. Then, in exchange (after washing 3x with PBS), the fixed SGN were incubated with a biotinylated anti-mouse antibody (Vector Lab) diluted (1:2,000) in 1.5% normal horse serum in PBS (Vectastain Elite ABC-Kit, Vector Labs, Burlingame, CA, USA) for 30 min at room temperature. After three washing steps with PBS, the SGN were incubated for 30 min at room temperature with the ABC complex solution (Vector Lab) to visualize the antigen-antibody-complex, followed by the peroxidase DAB substrate (Peroxidase Substrate Kit DAB, Vector Lab) for 10 min until the final washing occurred (PBS, 3x).

Using transmission light microscopy (Olympus CKX 41, Hamburg, Germany), cell counting was performed and was in the following set into relation to the seeding control (in %) for the determination of the survival rates (except for the initial screening experiment, where only the number of neurons has been determined). Surviving neurons, that showed immunostaining for the 200 kDa neurofilament and also had neurites with a length of at least three times the cell diameter (Gillespie et al., 2001), were counted as survived spiral ganglion neurons. To determine the length of the five longest neurites, a coupled CCD-camera (Colorview XS, SIS, Münster, Germany) and the image analyzing software CellP (SIS; Olympus) were used, resulting in five images of neurons in each well.

### Western Blot Analysis

For the western blot analysis spiral ganglia were isolated as described above (see “Spiral Ganglion Isolation and Cultivation” section until the dissociation step). The spiral ganglia were divided into three parts (basal, medial, and apical) and were cultivated as whole strands in a poly-D/L-ornithine and laminin-coated Nunc 48-well plate (Thermo Fisher Scientific, Waltham, MA, USA). We used spiral ganglia of three cochleae per condition. The spiral ganglia were incubated for 48 h in medium, medium supplemented with C3, and medium supplemented with C3 and BDNF. After 48 h the medium was removed and the spiral ganglia were washed with PBS. After removal of the PBS, the spiral ganglia were stored at −20°C.

Cells were scraped into Laemmli sample buffer. The obtained suspension was shaken at 37°C for 10 min. Ultrasonic disruption was performed in a cycle of 10 × 5 s, 5 × 10% sonic energy using a Sonotrode (Bandelin Electronic, Berlin, Germany). The lysate was then incubated at 95°C for 10 min.

Complete lysate proteins were separated using sodium dodecyl sulfate-polyacrylamide gel electrophoresis (SDS-PAGE) (Cti-Chemie u. Werkstoff-Technik GmbH, Idstein, Germany) and subsequently transferred onto nitrocellulose membranes by a tank blot system. The membranes were blocked with 5% (w/v) nonfat dried milk for 60 min; incubation with primary antibody was conducted overnight at 4°C and treatment with the secondary antibody at room temperature for 1 h. For western blot analysis, the following primary antibodies were used: RhoA was identified using a mouse monoclonal IgG from Santa Cruz Biotechnologies (Santa Cruz, CA, USA). ADP-ribosylated RhoA was detected by a specific antibody against ADP-ribosylated RhoA (ViF140_A1-hFc antibody was kindly provided by Viola Fühner and Michael Hust, Technische Universität Braunschweig, Germany; Rohrbeck et al., 2016). β-actin (Sigma–Aldrich Chemie GmbH, Munich, Germany) was used as the loading control. For the chemiluminescence reaction, electrochemiluminescence (ECL) Femto (Pierce, Thermo Fisher Scientific Inc., Rockford, IL, USA) was used.

### Statistical Analysis

All data were presented as mean values ± standard error of the mean (SEM). For statistical analysis, the software Graph Pad Prism was used and the significance of the obtained data was verified by applying the analysis of variance (ANOVA) test with Newman-Keuls multiple comparison test of all pairs. Only *p*-values of less than 0.05 were considered to be statistically significant. All data represent the mean of three independent approaches (N), including triplicates of each sample (n; *N* = 3, *n* = 3). Levels of significance are indicated as follows: n.s. = not significant; *p < 0.05; **p < 0.01; ***p < 0.001.

## Results

### Screening Experiments

**Figure 1:**
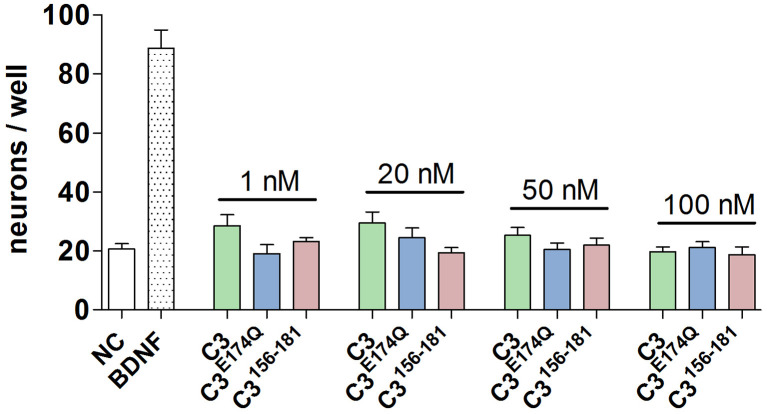
Screening of different concentrations and constructs of C3. The number of neurons per well is after the treatment with the three constructs of C3 at nanomolar concentrations (1 nM, 20 nM, 50 nM, and 100 nM) for 48 h on a comparable level to the negative control. Thus, the treatment of spiral ganglion neurons (SGN) with different constructs and concentrations of C3 is not toxic.

In initial screening experiments, the SGN were incubated for 48 h with various nanomolar concentrations (1, 20, 50, and 100 nM) of the three constructs of C3: the enzyme-competent wild-type (C3), an enzymatically inactive mutant (C3^E174Q^) generated through the exchange of the catalytic amino acid glutamate to glutamine at position 174 and a 26mer C-terminal peptide fragment covering amino acid 156–181 (C3^156-181^). None of the used concentrations of the tested C3 constructs were toxic or *per se* able to increase the survival rate of SGN (Figure 1). Based on these results and our previous studies with neuronal cells (Höltje et al., 2009; Loske et al., 2012), we decided to test the respective constructs of C3 at a concentration of 50 nM in the subsequent experiments.

### C3 Uptake From SGN

**Figure 2:**
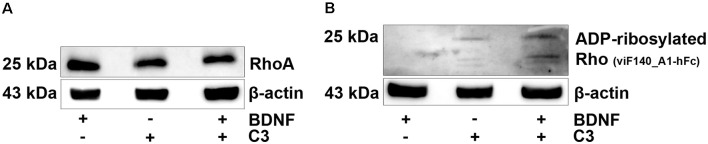
Western blotting experiments. Western blot analysis of cell lysates from SGN treated with brain-derived neurotrophic factor (BDNF), C3, and C3 plus BDNF. Cell lysates were prepared from SGN after 48 h cultivation. ß-actin served as the loading control. **(A)** RhoA could be detected in all samples, independent from the treatment. **(B)** After the treatment with C3 and C3 plus BDNF, ADP-ribosylated RhoA was detected in the SGN cell lysates. Thus, 50 nM of C3 was sufficient to ADP-ribosylate RhoA in intact SGN and confirmed uptake of C3.

First, we showed that RhoA is expressed by SGN cultures (Figure 2A). To confirm that C3 also entered SGN we performed additional western blot experiments to detect ADP-ribosylated RhoA in cell lysates of the *in vitro* cultured SGN. When C3 is taken up by cells, it ADP-ribosylated the RhoA protein and inactivated the Rho/ROCK pathway. Treatment with 50 nM of C3 was sufficient to ADP-ribosylate RhoA in intact SGN cell cultures (Figure 2B).

### Effects of C3 on the Survival Rate and Neurite Length of Spiral Ganglion Neurons *In vitro*

**Figure 3:**
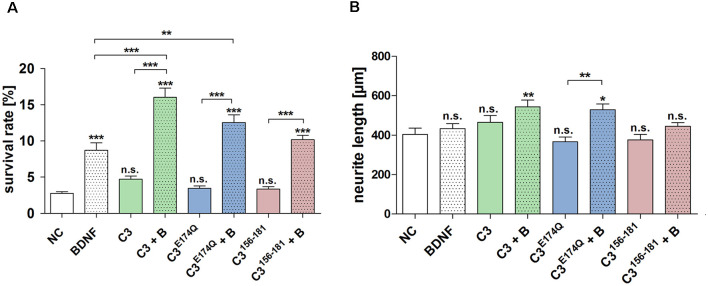
Effect of the different constructs of C3 exoenzyme on the survival rate and neurite length of SGN. **(A)** Treatment of SGN with C3, C3^E174Q^, or C3^156-181^ (50 nM) for 48 h did not increase the survival rate. Thus, only in combination with BDNF, the survival rates of SGN were significantly increased when compared to the negative control (NC; medium control). This effect was also detected when compared to its respective C3 construct alone. The combination of C3 and C3^E174Q^ with BDNF significantly increased the survival rate of SGN when compared to the BDNF control (50 ng/ml). **(B)** The neurite length of SGN did not show a significant increase when treated with the C3 alone. However, the combination of C3 and BDNF resulted in a significantly enhanced neurite length with *p* < 0.01 when compared to the control. This condition obtained the maximum of all measured neuronal lengths. The enzymatically inactive mutant C3^E174Q^ showed in combination with BDNF a significantly increased neurite length when compared to its pure application. Asterisks over the error bars represent the respective significance when compared to the NC, either for the survival rate or for the neurite length. NC, negative control; BDNF/B, brain-derived neurotrophic factor (BDNF); n.s., not significant.

**Figure 4:**
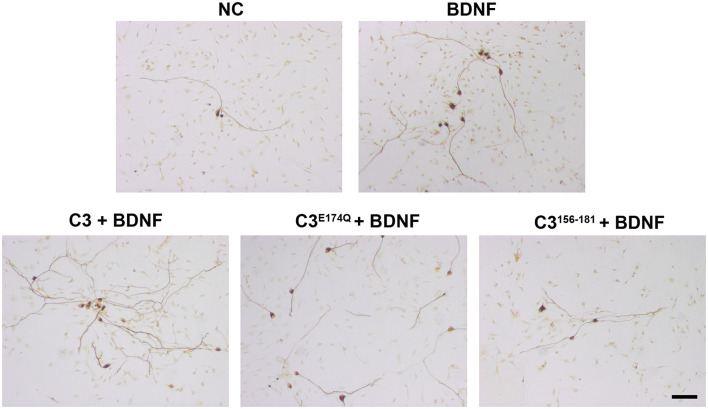
Representative images of SGN treated with various C3 constructs. Cultured SGN were treated with culture medium (NC), BDNF, C3 + BDNF, C3^E174Q^ + BDNF, and C3^156–181^ + BDNF for 48 h and were stained for 200 kDa neurofilament. The combination of all tested C3 constructs and BDNF resulted in more survived SGN when compared to BDNF only or culture medium only. Scale bar, 50 μm.

The treatment with C3, C3^E174Q^, or C3^156-181^ for 48 h did not significantly increase the survival rate of SGN (Figure 3A). However, the combination of the different C3 constructs with BDNF showed highly significantly increased neuronal survival rates when compared to NC. Representative microscopic images of the different treated SGN are depicted in Figure 4. The treatment of SGN with C3 plus BDNF resulted in the highest survival rate (16.03% ± 1.27%) and was highly statistically significant when compared to the BDNF control (SGN treated with BDNF (50 ng/mL); survival rate: 8.70% ± 1.04%, *p* < 0.001). The survival rate of SGN was also significantly increased after the cultivation with C3^E174Q^ in combination with BDNF in comparison to the BDNF control; survival rate 12.57% ± 1.04%, *p* < 0.01. Treatment with C3^156-181^ and BDNF (survival rate 10.19% ± 0.61%) did not significantly increase the survival rate of SGN when compared to BDNF alone. When compared to the negative control (NC; SGN without any treatment, survival rate: 2.76% ± 0.24%) the addition of the different C3 constructs alone did not show any significant enhancement of the survival rates, whereas all three combinations (C3 constructs + BDNF) showed significantly increased survival rates (*p* < 0.001). In each group, the survival rate of the combination was strongly increased when compared to the respective C3 alone (*p* < 0.001).

The combined treatment with C3 plus BDNF (544.1 μm ± 33.88 μm; *p* < 0.01) and C3^E174Q^ plus BDNF (529.6 ± 28.96 μm; *p* < 0.05) significantly enhanced the neurite length when compared to the negative control (Figure 3B, NC 404.1 μm ± 31.37 μm). A significantly increased neurite length was observed for C3^E174Q^ plus BDNF when compared to C3^E174Q^ alone (529.6 μm ± 28.96 μm and 366.6 μm ± 23.76 μm respectively; *p* < 0.01). Treatment with the other C3 constructs in combination with BDNF did not result in a significant increase in neurite length.

### Inhibition of the Neuroprotective Effect of BDNF/C3

**Figure 5:**
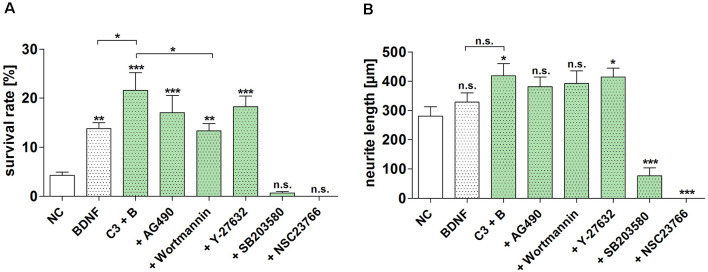
Inhibition of the neuroprotective effect of different C3 constructs on SGN. The determined survival rate and measured neurite length of SGN treated with C3 wild-type in combination with BDNF and different selected inhibitors for 48 h are depicted. **(A)** Treatment with C3 plus BDNF significantly increased the survival rate of SGN in comparison to the negative control and BDNF alone. Additional application of wortmannin significantly decreased the survival rate when compared to C3/BDNF treatment alone. The inhibitors SB203580 and NSC23766 significantly decreased the survival rate and neurite length of SGN after the treatment with C3 wild-type in combination with BDNF. **(B)** The measured neurite length of SGN was significantly increased after the treatment with C3 plus BDNF and C3 plus BDNF in combination with Y-27632 when compared to the negative control. Asterisks over the error bars indicate the respective significance to the NC. NC, negative control; C3, 50 nM; BDNF/B, brain-derived neurotrophic factor, 50 ng/ml; SGN, spiral ganglion neurons (SGN); n.s., not significant.

Since the neuroprotective effect of BDNF and C3 wildtype was the most promising of the tested constructs, we performed the inhibitor experiments only with this C3 construct. The SGN were treated with the different inhibitors and C3/BDNF for 48 h. Based on recently published data we have chosen two inhibitors to remove the mediated effect of C3: NSC23766 inactivating Rac1 (von Elsner et al., 2016) and SB203580 (p38) (von Elsner et al., 2017). To verify that the observed neuroprotective effects are due to the application of C3 we used AG490 (inhibitor of the JAK/STAT pathway) and wortmannin (PI3K inhibitor) to remove the effect of BDNF. Additionally, we used Y-27632 inhibiting ROCK to increase the inhibitory effect of C3 on the Rho signaling pathway. The highest survival rate of C3 and BDNF was 21.57% ± 3.65% and was statistically significantly higher when compared to the negative control (NC, *p* < 0.001) and to BDNF alone (Figure 5A; *p* < 0.05). Combinations of C3/BDNF and AG490 and C3/BDNF and Y-27632 significantly increased the survival rate when compared to the negative control (*p* < 0.001), but not when compared to C3/BDNF or BDNF alone. But treatment with C3/BDNF and wortmannin significantly decreased the survival rate of SGN when compared to C3/BDNF alone (Figure 5A; *p* < 0.01). The observed survival rate of C3/BDNF and wortmannin is on a comparable level to BDNF alone indicating that the potentiated C3 effect has been removed. The neuroprotective effect of C3 and BDNF was almost completely and significantly removed by the inhibitors SB203580 and NSC23766 (Figure 5A; *p* < 0.001). Treatment with NSC23766 (inhibition of Rac1) inhibited the outgrowth of neurites from SGN in the cultures (Supplementary Figure 1). According to Gillespie et al. (2001), surviving neurons are defined as 200 kD neurofilament positive cells with neurites of at least three times the soma diameter in length. However, SGN in the cultures treated with NSC23766 displayed no outgrown neurites, but the somata of several neurons were stained for neurofilament (Supplementary Figure 1, arrowhead). Thus, the survival rate for NSC23766 was zero, although several somata stained regularly but were not counted as surviving neurons. After the treatment with SB203580, some SGN survived (experiment 1: five neurons in total, experiment 2: 14 neurons in total, and experiment 3: four neurons in total) and several cell somata without neurites were additionally detectable. This results in a survival rate of 0.66% ± 0.3% for SB203580. Thus, the two inhibitors were not toxic for the cells but inhibited even the spontaneous outgrowth of neurites that can be observed in the negative controls (NC).

The neurite length of SGN was significantly increased after the combined treatment of C3 plus BDNF as well as after the treatment with C3 plus BDNF and the inhibitor Y-27632 when compared to the negative control (NC; Figure 5B; *p* < 0.05). The neurite length was significantly decreased when SGN were treated with SB203580 and NSC23766 (for SB203580 the mean neurite length was 77.05 μm ± 26.93 μm, *p* < 0.001; NSC23766 showed no survived SGN, *p* < 0.001). The treatment with the inhibitors wortmannin and AG490 did not significantly influence the neurite length when compared to the negative control.

## Discussion

Rho-GTPases are involved in different cellular functions such as transcriptional activation, transmembrane signaling, and regulation of the actin cytoskeleton (Ahnert-Hilger et al., 2004). In the inner ear, noise-induced trauma leads to outer hair cell death and this is mediated by the activation of Rho-GTPases (Chen et al., 2012). Adenosine diphosphate- (ADP-) ribosyltransferases target Rho-GTPases and thereby exert diverse effects (von Elsner et al., 2016). ADP-ribosylation describes the process of transferring an ADP-ribosyl group from NAD^+^ to a protein by an ADP-ribosyltransferase (Pallen et al., 2001). Bacterial exotoxins are known to use this molecular reaction to modulate eukaryotic cells. C3 exoenzyme from *Clostridium botulinum* belongs to the group of ADP-ribosyltransferases and inactivates selectively RhoA, B, and C by coupling ADP-ribose moiety. The action of different constructs of C3 on SGN *in vitro* have been investigated in the present study: an enzymatically inactive mutant (C3^E174Q^) through the exchange of the catalytic amino acid glutamate to glutamine on position 174, an enzymatically inactive amino-acid fragment covering amino acid 156–181 (C3^156-181^) and the full-length C3. Our results show that all three different constructs of the C3 exoenzyme derived from *Clostridium botulinum* increase the neuroprotective effect of BDNF where only C3 and C3^E174Q^ significantly increased the effect in comparison to BDNF alone.

The C3 exoenzyme from *Clostridium botulinum* (C3) is a single-domain mono-ADP-ribosyltransferase with important structural motifs including the ADP-ribosylation toxin-turn-turn (ARTT) loop which is involved in protein substrate recognition and harbors the catalytic amino acid residue E174; the phosphate-nicotinamide loop (PN-loop) which is involved in the binding of NAD^+^ and the STS-motif (serine-threonine–serine) which is thought to maintain the reaction cavity (Han et al., 2001; Ménétrey et al., 2008). Mutation of the catalytic amino acid residue (E174) of C3 cuts down transferase activity and blocks modification of Rho. The 26mer peptide is the extended ARTT-loop of C3 and covering the region of the catalytic amino acid E174 (C3^156–181^ peptide) and the amino acid responsible for binding to the protein substrate Rho (F169) reside in the ARTT loop. The ADP-ribosylation of Rho does not seem to be the decisive step, since both enzymatically deficient C3^E174Q^ and 26mer C3 peptide have neurotrophic properties and have an axonotrophic effect on primary neurons of murine and human origin (Loske et al., 2012). Enzyme-deficient C3^E174Q^ and C3^156–181^ peptides do not act on astrocytes (Höltje et al., 2008) and microglia (Hoffmann et al., 2008). Here only the enzymatically active C3 induces the release of pro-inflammatory mediators from microglia, thus this effect seems to be Rho-dependent.

This is especially of interest since C3 acts on a different molecular level than BDNF or other classical growth factors that bind to their specific receptors for the activation of distinct intracellular signaling pathways. Despite the lack of a receptor-binding domain, several studies showed the uptake of C3 suggesting an intracellular target for pharmacological activity (Rotsch et al., 2012; Rohrbeck et al., 2015b; Adolf et al., 2016). We could also confirm the uptake of C3 by SGN cultures *via* detecting ADP-ribosylated RhoA in cell lysates. All three constructs of C3 showed axonal and functional recovery indicating that the neurotrophic effect is enzyme-independent and the functional activity of the ADP-ribosyltransferase of C3 is not necessary for the axonal and dendritic growth (Ahnert-Hilger et al., 2004; Höltje et al., 2009; Huelsenbeck et al., 2012; Loske et al., 2012). Similar effects have been observed in neurons from other organ systems. For example, nanomolar concentrations (50 nM) of C3 and the enzymatically inactive mutant full-length C3^E174Q^ promote axonal growth in hippocampal primary cultures (Ahnert-Hilger et al., 2004). The peptide C3^156–181^ also promoted neuronal regenerative capacity after spinal cord lesions in the mouse and in a lesion model of the rat sciatic nerve (Huelsenbeck et al., 2012). The axonotrophic effect of C3 seems to be independent of RhoA inhibition and is weaker, but reminiscent of those induced by neurotrophic factors (Auer et al., 2012). In our *in vitro* model, different nanomolar concentrations of C3 alone failed to protect serum-deprived SGN from apoptosis. However, the well-known neuroprotective effect of BDNF (50 ng/ml) is significantly potentiated by the simultaneous application of C3. The inactivation of RhoA resulted in an activation of the growth and repair mechanisms of neuronal cells. Interestingly, the Rho proteins can be pro-apoptotic or anti-apoptotic, depending on the cellular conditions and the cell types, respectively (Kanekura et al., 2005). Rho-GTPases are activated by laminin thereby mediating neurite outgrowth (Brors et al., 2003). The inhibition of the Rho-GTPases Rac and Cdc42 by the *Clostridium difficile* toxin B reduced neurite outgrowth from SGN (Brors et al., 2003). In our study, the inhibition of Rac1 (NSC 27632) also abolished the outgrowth of neurites in SGN.

Neurotrophins such as NT-3 and BDNF mediate their effect by the stimulation of its corresponding neurotrophin receptor (TrkC, TrkB) and Rho inhibitors do not reduce NT-3 mediated neurite outgrowth (Brors et al., 2003). Thus, neurotrophin signaling of NT-3 seems to be mediated *via* a pathway that is independent of Rho-GTPases (Rac, Cdc42, and Rho; Brors et al., 2003). The binding of mature neurotrophins to the low-affinity neurotrophin receptor p75 (p75NTR) has been shown to inactivate RhoA and thereby promoting neurite outgrowth (Yamashita et al., 2002; Koizumi et al., 2020). The binding of pro-neurotrophins to the p75NTR activates RhoA resulting in growth cone collapse and inhibition of axon growth (Kalpachidou et al., 2019). However, evidence exists for the inner ear that p75NTR is also involved in the formation and regeneration of neurites (Inoue et al., 2014). The exogenous application of mature BDNF in our study could induce binding of mature BDNF to TrkB and also to p75NTR since it exists an oversupply of mature BDNF. Treatment with proBDNF alone did not increase the survival rate or neurite length of SGN in our *in vitro* cell culture model, whereas a combined treatment of mature BDNF and proBDNF only increased the survival rate of SGN when the two neurotrophin forms were balanced or mature BDNF is overbalanced (Schulze et al., 2020). Furthermore, inhibition of Rho-GTPases protects hair cells from aminoglycoside toxicity *in vitro* (Bodmer et al., 2002). Growth factors such as FGF-2 or NGF induce a decrease of RhoA GTP levels *via* the activation of PI3K which in turn activates Rac activity inducing an inactivation of RhoA (Auer et al., 2012). Indeed, inhibition of PI3K by using the wortmannin inhibitor abolished the increment induced by C3 on top of BDNF. This possibly indicates that PI3 kinase is involved in the additional response when C3 is added in cultures stimulated by BDNF.

**Figure 6:**
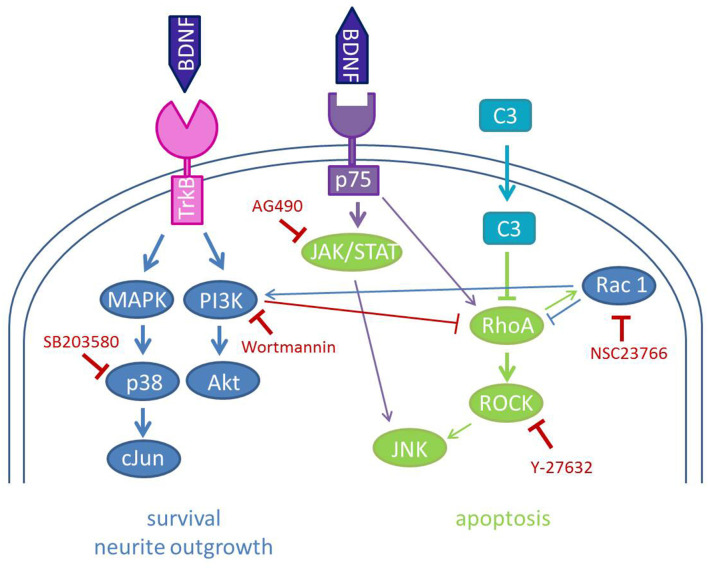
Schematic illustration of the used inhibitors and the signal transduction pathways involved in BDNF and C3 effects on SGN. The neurotrophin brain-derived neurotrophic factor (BDNF) binds to its high-affinity receptor TrkB and its low-affinity receptor p75NTR. The main interactions of BDNF are depicted in blue and mediate neurite outgrowth and survival. Binding to p75NTR leads to the activation of the JAK/STAT pathway and also of RhoA inducing apoptosis and growth inhibition. C3 inhibits the activity of RhoA which results in the inactivation of the ROCK signaling pathway and diminishes growth inhibition. Rac1 activates the PI3K to activate Akt and thereby enhances neuronal survival. The used inhibitors of this study are depicted in red. Inhibition of Rac1 by NSC23766 and p38 by SB203580 leads to no outgrown neurites in SGN. Wortmannin (inhibition of PI3K) seems also to reduce the survival rate of SGN after combined treatment with C3 and BDNF.

Our results suggest an additive effect of BDNF and C3, including its enzymatically inactive mutant (C3^E174Q^). Thus, mechanisms other than Rho inhibition may be involved. Among the inhibitors of intracellular pathways used in the present study (for an overview see Figure 6), the p38MAPK inhibitor and the Rac1 inhibitor abolished the protective effects observed by the treatment with BDNF and C3. It has been shown that the phosphorylation of the direct upstream MAPK kinase of JNK is not inhibited by C3 (von Elsner et al., 2017). Activation of all JNK isoforms has been shown to mediate neurite outgrowth in SGN (Atkinson et al., 2011). Inhibition of p38MAPK (SB203580) results in a reduction of the survival rates and the neurite outgrowth in BDNF/C3 treated primary auditory neurons (i.e., SGN; Figure 5). There is a body of evidence that p38 MAP kinase is essential for neurite outgrowth (Morooka and Nishida, 1998; Takeda and Ichiijo, 2002). However, the underlying molecular mechanisms are still unknown. A similar effect was observed for the selective Rac1 inhibitor NSC23766. After the inhibition of Rac1, the outgrowth of neurites was suppressed and therefore no SGN were defined as surviving (neurites with a length of three times the soma diameter) in the cultures. Therefore we checked in an additional experiment whether inhibition of Rac1 without a C3 treatment resulted in reduced SGN survival. In cultures treated with BDNF which is necessary for the survival of SGN in cell culture systems, we could not detect any surviving SGN but somata without neurites as well as glial cells and fibroblasts (Supplementary Figure 1). C3 reduces the activity of c-Jun and JNK only in the presence of p38α and this effect was not observed in p38α-deficient cells (von Elsner et al., 2017). In neonatal cochlear spiral ganglion explants, neurite formation is induced by BDNF *via* the p38 signaling pathway (Mullen et al., 2012). Thus, in the presence of BDNF and C3, neurite outgrowth may not be mediated by downstream activation of c-Jun and JNK, but by alternative mechanisms. Neuronal survival, as well as neurite outgrowth in SGN, is mediated *via* PI3K/Akt (Mullen et al., 2012). Interestingly, wortmannin, an inhibitor of PI3K, significantly reduced survival in SGN treated with BDNF and C3 to the level achieved by BDNF treatment alone. Since RhoA inhibits Rac and Cdc42 in neurons thereby inhibiting neurite outgrowth, inhibition of RhoA by C3 should reverse this inhibition. In SGN, combined treatment with C3 and BDNF leads to significantly enhanced neurite outgrowth. Since the Rac1 inhibitor (NSC23766) prevents the outgrowth of new neurites from SGN completely without affecting the proliferation of bystander cells such as fibroblasts or glial cells, no surviving SGN and neurite outgrowth can be determined. Thus, Rac1 is essentially required for neuroregeneration (Jaffe and Hall, 2005) acting together with Cdc42 as positive regulators of neurite outgrowth (Luo, 2000; Auer et al., 2011) and formation of neurite extensions (Estrach et al., 2002). Moreover, Rac stimulates the PI3K/Akt pathway that induces pro-survival and inhibits apoptotic signals (Stankiewicz and Linseman, 2014). Linseman and colleagues showed also that inactivation of Rac1 GTPase induced apoptosis of primary central ganglion neurons which could explain our observations with the treatment with the Rac1 inhibitor (Stankiewicz and Linseman, 2014). Lingor and colleagues combined Y-27632 and ciliary neurotrophic factor (neuroprotective factor in SGN culture as well Schwieger et al., 2015) and showed a more effective neurite growth and regeneration of retinal ganglion cells (Lingor et al., 2008). Additionally, the combination of BDNF and Y-27632 enhanced the neurite length of SGN *in vitro* (Kramer et al., 2017). Inhibition of ROCK by Y-27632 increased the number of axons projecting to the hair cells as well as the number of synapses in an organotypic culture of the cochlea (Koizumi et al., 2020). A ROCK inhibitor (H-1152) increased the neurite outgrowth of SGN (Lie et al., 2010).

The utilization of C3 as a neuroregenerative agent is limited by poor cell membrane permeability. The intermediate filament protein vimentin acts as an interaction partner between C3 and the cell membrane of different cell lines and primary neuronal cells thereby mediating internalization of C3 (Rohrbeck et al., 2014, 2017; Adolf et al., 2016). The membrane-bound β1-integrin is an additional interaction partner for C3 (Rohrbeck et al., 2017; Adolf et al., 2019). Thus, the interaction of specific cells (e.g., neurons) with C3 might aid in mediating effects despite limited entrance into the cells to interact with Rho. Enzyme-deficient variants of C3 are as effective in our *in vitro* model and in other studies as C3 (Just et al., 2011; Huelsenbeck et al., 2012; Rohrbeck et al., 2014, 2015a,b). The C-terminal peptide fragment covering amino acids 154–182 of C3 has been shown to promote dendritic and axonal growth and branching as well as increased synaptic connectivity in hippocampal neurons (Höltje et al., 2009). Peptide fragments derived from C3 enabled recovery from spinal cord injury in mice (Boato et al., 2010). In the present study, C3^156–181^ in addition to BDNF improved neuronal survival. The neuroprotective effects of C3 have also been demonstrated in a model of NMDA-induced excitotoxicity in the retina (Wang et al., 2014). Protection and regeneration of SGN is one major goal in otology-related research: these neurons degenerate over time after the loss of hair cells and are the target cells for hearing rehabilitation with cochlear implants. Neuronal health seems to be associated with improved hearing outcomes in cochlear implantation (Seyyedi et al., 2014; DiNino et al., 2019). Moreover, lentivirus-mediated C3 expression lowered intraocular pressure in an animal model (Tan et al., 2018b). Neuroprotection and reduction of pressure could be of clinical importance in humans suffering from Menières disease, a condition that leads to hearing loss and is characterized by inner ear hydrops.

## Conclusion

The therapeutic potential of factor combinations such as BDNF and C3 are of great importance for the development of drugs for the treatment of inner ear diseases. In the present study, we were able to show the neuroprotective potential of C3 in combination with BDNF on primary inner ear specific cells (spiral ganglion neurons). These results are very promising, but the observed effect needs to be further validated in an *in vivo* model, e.g., noise-trauma model.

## Data Availability Statement

The original contributions presented in the study are included in the article/Supplementary Material, further inquiries can be directed to the corresponding author.

## Author Contributions

JH, AR, and AW designed the research. MS contributed to the initial experiments. LH performed the experiments and analyzed the data. JH, LH, and AW wrote the manuscript. JH and LH contributed equally. IJ and TL provided administrative support. All authors contributed to the article and approved the submitted version.

## Conflict of Interest

The authors declare that the research was conducted in the absence of any commercial or financial relationships that could be construed as a potential conflict of interest.
