# Corrigendum: Potentiation of brain-derived neurotrophic factor-induced protection of spiral ganglion neurons by C3 exoenzyme/Rho inhibitor

**DOI:** 10.3389/fncel.2022.1078962

**Published:** 2022-11-03

**Authors:** Jennifer Harre, Laura Heinkele, Melanie Steffens, Athanasia Warnecke, Thomas Lenarz, Ingo Just, Astrid Rohrbeck

**Affiliations:** ^1^Department of Otorhinolaryngology, Head and Neck Surgery, Hannover Medical School, Hannover, Germany; ^2^Cluster of Excellence “Hearing4all” of the German Research Foundation (EXC 2177/1), Hannover, Germany; ^3^Institute of Toxicology, Hannover Medical School, Hannover, Germany

**Keywords:** spiral ganglion neurons, RhoA, C3 exoenzyme, brain-derived neurotrophic factor, neuroprotection

In the published article, there was an error in the author list, and authors “Jennifer Harre and Laura Heinkele” were erroneously not marked as equally contributing and sharing first authorship. The corrected author list appears below.

“Jennifer Harre^1, 2*^^†^, Laura Heinkele^1^^†^, Melanie Steffens^1^, Athanasia Warnecke^1, 2^, Thomas Lenarz^1, 2^, Ingo Just^3^ and Astrid Rohrbeck^3^”

This also affects the **Author contributions** section within the published article. The corrected **Author contributions** section is below.

## Author contributions

JH, AR, and AW designed the research. MS contributed to the initial experiments. LH performed the experiments and analyzed the data. JH, LH, and AW wrote the manuscript. JH and LH contributed equally. IJ and TL provided administrative support. All authors contributed to the article and approved the submitted version.

The authors apologize for this error and state that this does not change the scientific conclusions of the article in any way. The original article has been updated.

## Publisher's note

All claims expressed in this article are solely those of the authors and do not necessarily represent those of their affiliated organizations, or those of the publisher, the editors and the reviewers. Any product that may be evaluated in this article, or claim that may be made by its manufacturer, is not guaranteed or endorsed by the publisher.

