# Supplementary figures and images for: Potentiation of Brain-Derived Neurotrophic Factor-Induced Protection of Spiral Ganglion Neurons by C3 Exoenzyme/Rho Inhibitor
Source: Front Cell Neurosci. 2021 Mar 11;15:602897. doi: 10.3389/fncel.2021.602897 (PMC7991574; doi:10.3389/fncel.2021.602897)

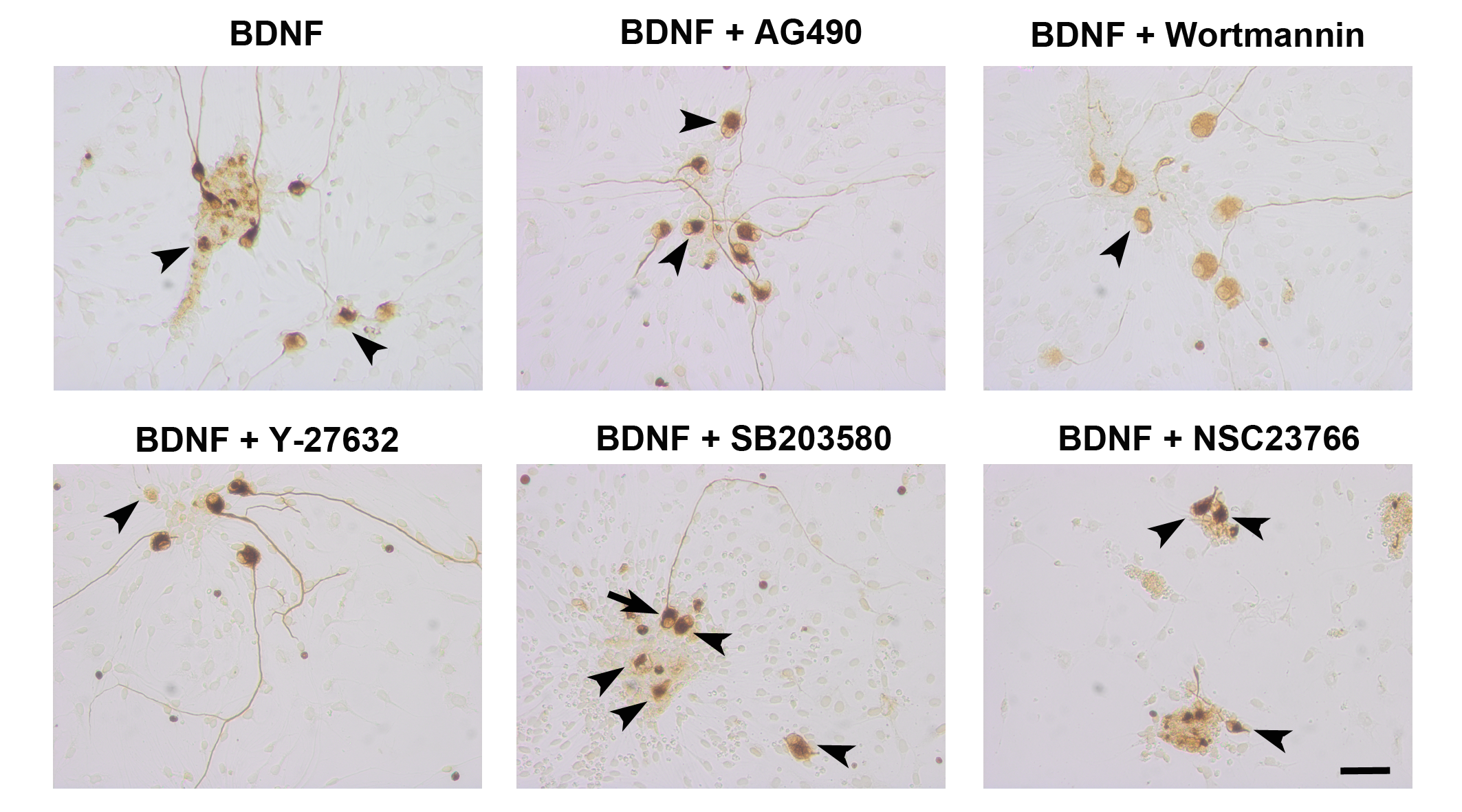

Supplement: Supplementary file 1 [file Image_1.TIF]
